# The Book World of Medicine and Science

**Published:** 1901-08-17

**Authors:** 


### The Book World of Medicine and Science.

The Law Relating to Poor Law Medical Service and Vaccination. By M. Greenwood, M.D., LL.B. Published under the direction of the Poor Law Medical Officers' Association of England and Wales. (London: Baillière, Tindall, and Cox. 1901. Price 2s. 6d. net.)

It probably is not until after a medical man has obtained a poor law appointment that he becomes fully aware of the responsibility of his position. He has become part and parcel of the machinery established by law and covering the whole of the country, and unless he thoroughly understands his position, his duties, and his legal relations with the poor and with his fellow officials, he is sure before long to put his foot in it somehow or another. We cannot but feel that many of the misunders tandings and apparent antagonisms which we so often hear of between the medical and other officials under the poor law might have been prevented if medical men had been more careful than they sometimes seem to be in studying the obligations of all concerned as laid down by law. But this study has hitherto been by no means an easy matter to accomplish. The books on the subject have been expensive and lately somewhat out of date. Although the poor law is old enough, a great deal of its administration is regulated by Orders which are issued from time to time, so that to obtain any correct idea of his obligations a medical officer must consider both the law and the Orders, and must wade through much literature which is often unattainable. We therefore welcome this small and well digested handbook, which by the sanction of the Association by which it is issued lays down in a somewhat authoritative way all that the poor law medical officer, be he district or workhouse medical officer, or public vaccinator, needs to know on the subject. It is most carefully arranged, and being written by one who is a barrister as well as a medical practitioner, it may be regarded as reliable from a legal as well as a medical point of view. A good deal that is said about the grave defects which exist in the Poor Law Officers' Superannuation Act will be read with interest. Dr. Greenwood points out that the effect of the Act is such that each union has the strongest inducement to treat their officers harshly in their superannuation claims, as by so doing they keep down their local rates. "Hence there is a tendency on the part of Boards of Guardians to avail themselves of any pretext to get rid of old officers when the time for superannuation draws nigh." It also results that Poor Law officers who have lost their posts are at a great disadvantage in getting others, especially if they have held the former for many years and their time for retirement is approaching. The law presses peculiarly heavily upon doctors in the country, for in many places it is impossible for the medical officer to reside within his district. He is therefore subject to re-election annually or triennially, and at any of these re-elections it is always open to the guardians to pass him over in favour of another candidate. Were there a central fund it would remove the inducement to act in this way. A full account is given of the duties and responsibilities of the public vaccinator, and the law on the subject is clearly laid down and is illustrated by examples. Take it altogether we regard this book as valuable in itself, and all the more valuable since it fills a very obvious gap in medical literature.

Local London: A Municipal Directory for the Metropolis and Suburbs, 1901-2. (Westminster: P. S. King and Son. 1901. Price 6d. net.)

This is a most admirable little book. It gives a large amount of information about London and its widely extending suburbs, information which could not be otherwise obtained without consulting numerous authorities which would be by no means easy to lay one's hands upon. No doubt, in regard to the municipalities of inner London, various handbooks are available, but we know of no other book which contains as this does within its modest covers full details relating to all the municipal affairs not only of the City and the County of London, but also of some sixty adjacent, outside, self-governing areas. The general plan of the work is to give in regard to the more important municipalities the names and addresses of the mayor, aldermen, and councillors, with a list of the officials and of the public municipal institutions, while in regard to the smaller suburban districts the details given are not so full Full information is also given as to the County Council, the School Board, metropolitan boards of guardians, and a considerable number of public bodies, such as the Metropolitan Asylums Board, the Thames Conservancy, etc. A map which particularly appeals to us as showing what a complicated place London is, is one giving the boundaries of London according to six different definitions, viz., County of London, Criminal Court, Police Courts, Poor Law and Registration, Police area, and Postal London, together with the various county boundaries which are included within the area of Greater London. The only two of these boundaries which appear to coincide throughout are those of the County of London and of the Poor Law London; all the others, although they touch here and there, soon wander off again, so that none of the areas they enclose are alike. This little handbook is a most useful and well-arranged publication.

Difficult Labour: A Guide to its Management, for Students and Practitioners. By Ernest Herman, M.B.Lond., F.R.C.P. New and revised edition. (London: Cassell and Co. 1901. Price 12s. 6d.)

During the six or seven years that this book has been in the hands of the profession it has taken a high position as a guide in obstetric difficulties. Putting on one side the temptation to write a complete treatise on midwifery, Dr. Herman set himself the task of stating, with just that tinge of dogmatism which comes well from a teacher of experience, what should be done in the various difficulties with which the practitioner of midwifery has occasionally to cope and always to be prepared for. Presuming that the reader knew all about the elementary aspects of the subject he devoted himself to the difficult cases only, and by so doing produced just the book which the practitioner wanted. This edition has been revised throughout, but the most important alterations are those which have reference to the technique of Cæsarian section and symphysiotomy. The whole book is thoroughly up to date and is written in a style which makes it interesting as well as instructive, while the advice given is sound and evidently the outcome of a ripe experience. This is we think the highest praise.

